# A nonrandomized trial of vitamin D supplementation for Barrett’s esophagus

**DOI:** 10.1371/journal.pone.0184928

**Published:** 2017-09-18

**Authors:** Linda C. Cummings, Prashanthi N. Thota, Joseph E. Willis, Yanwen Chen, Gregory S. Cooper, Nancy Furey, Beth Bednarchik, Bronia M. Alashkar, John Dumot, Ashley L. Faulx, Stephen P. Fink, Adam M. Kresak, Basel Abusneineh, Jill Barnholtz-Sloan, Patrick Leahy, Martina L. Veigl, Amitabh Chak, Sanford D. Markowitz

**Affiliations:** 1 Department of Medicine, University Hospitals Cleveland Medical Center, Cleveland, Ohio, United States of America; 2 Department of Medicine, Case Western Reserve University, Cleveland, Ohio, United States of America; 3 Case Comprehensive Cancer Center, Cleveland, Ohio, United States of America; 4 Medical Service, Louis Stokes Cleveland Veterans Affairs Medical Center, Cleveland, Ohio, United States of America; 5 Digestive Diseases Institute, Cleveland Clinic, Cleveland, Ohio, United States of America; 6 Department of Pathology, University Hospitals Cleveland Medical Center, Cleveland, Ohio, United States of America; 7 Department of Pathology, Case Western Reserve University, Cleveland, Ohio, United States of America; 8 William T. Dahms Clinical Research Unit, University Hospitals Cleveland Medical Center, Cleveland, Ohio, United States of America; 9 Division of General Medical Sciences, Case Western Reserve University, Cleveland, Ohio, United States of America; University Hospital Llandough, UNITED KINGDOM

## Abstract

**Background:**

Vitamin D deficiency may increase esophageal cancer risk. Vitamin D affects genes regulating proliferation, apoptosis, and differentiation and induces the tumor suppressor 15-hydroxyprostaglandin dehydrogenase (PGDH) in other cancers. This nonrandomized interventional study assessed effects of vitamin D supplementation in Barrett’s esophagus (BE). We hypothesized that vitamin D supplementation may have beneficial effects on gene expression including 15-PGDH in BE.

**Methods:**

BE subjects with low grade or no dysplasia received vitamin D_3_ (cholecalciferol) 50,000 international units weekly plus a proton pump inhibitor for 12 weeks. Esophageal biopsies from normal plus metaplastic BE epithelium and blood samples were obtained before and after vitamin D supplementation. Serum 25-hydroxyvitamin D was measured to characterize vitamin D status. Esophageal gene expression was assessed using microarrays.

**Results:**

18 study subjects were evaluated. The baseline mean serum 25-hydroxyvitamin D level was 27 ng/mL (normal ≥30 ng/mL). After vitamin D supplementation, 25-hydroxyvitamin D levels rose significantly (median increase of 31.6 ng/mL, p<0.001). There were no significant changes in gene expression from esophageal squamous or Barrett’s epithelium including 15-PGDH after supplementation.

**Conclusion:**

BE subjects were vitamin D insufficient. Despite improved vitamin D status with supplementation, no significant alterations in gene expression profiles were noted. If vitamin D supplementation benefits BE, a longer duration or higher dose of supplementation may be needed.

## Introduction

Esophageal adenocarcinoma (EAC) has risen markedly in incidence in the United States over the last several decades.[1, 2] Risk factors for EAC include its precursor lesion, Barrett’s esophagus (BE); gastroesophageal reflux disease;[3] male gender;[4] and obesity.[5] EAC typically develops through a stepwise progression from metaplasia to dysplasia to adenocarcinoma; cellular proliferation has also been correlated with neoplastic progression. Given the poor prognosis of EAC with a 5-year relative survival rate of 20% overall and 40% even when it is localized,[6] prevention would be ideal. Prevention through endoscopic ablation of BE reduces the risk of EAC but does not completely eliminate it and can be complicated albeit rarely by recurrent dysplasia, strictures, or buried BE glands beneath neosquamous epithelium.[7] In light of these limitations, chemoprevention is an appealing approach.

Previous chemoprevention studies in the field have focused on non-steroidal anti-inflammatory drugs (NSAIDs), which are associated with reduced EAC incidence in BE patients.[8–10] NSAIDs inhibit prostaglandins through suppression of cyclooxygenase-1 (COX-1) and cyclooxygenase-2 (COX-2),[11] the latter of which is overexpressed in BE and EAC.[12] Prostaglandin E2 (PGE2), a pro-inflammatory prostaglandin regulated by the COX-2 pathway, has been associated with proliferation[13] and cellular migration[14] in BE and EAC. Although short-term treatment with high dose aspirin, a COX inhibitor, in combination with the proton pump inhibitor (PPI) esomeprazole decreases BE mucosal PGE2 levels,[15] chronic NSAID use can be complicated by gastrointestinal toxicity. Celecoxib, a COX-2 inhibitor, was not found to prevent progression of dysplastic BE to EAC,[16] and also may raise the risk of cardiovascular disease.[17]

An alternative chemopreventive approach is to target Hydroxyprostaglandin Dehydrogrenase 15-(NAD) (Entrez Gene name HPGD/Gene ID 3248, also known as 15-PGDH) to increase PGE2 breakdown. PGDH is a tumor suppressor involved in the degradation of PGE2 and, thus, is a direct antagonist of the COX-2 pathway of PGE2 production. Low 15-PGDH levels are associated with progression of bladder cancer[18] and gastric cancer,[19] and resistance to the chemopreventive effects of celecoxib in the colon.[20] In the esophagus, low 15-PGDH levels are associated with EAC[21] and high grade dysplasia (HGD) in BE.[22] Restoration of 15-PGDH expression suppresses growth of esophageal cancer cell lines.[21] Vitamin D has been shown to induce 15-PGDH in some cancers.[23, 24] Vitamin D is a group of prohormones, including vitamin D_2_ (ergocalciferol) and vitamin D_3_ (cholecalciferol), integrally involved in calcium homeostasis and bone metabolism. Vitamin D, in its active form as calcitriol, binds vitamin D receptor (VDR) to form a complex that acts as a transcription factor, regulating multiple downstream pathways involved in proliferation, apoptosis, and differentiation. Calcitriol and its analogs exhibit anti-proliferative effects in various cancer cell lines.[25, 26] Vitamin D deficiency has been associated with insulin resistance, obesity,[27] and increased risk for esophageal cancer.[28] We hypothesized that vitamin D supplementation may have beneficial effects on gene expression (including 15-PGDH expression) in BE. Our primary aim was to assess the effects of vitamin D supplementation on 15-PGDH expression in BE. Secondary aims were to evaluate the effects of vitamin D supplementation on vitamin D status and global gene expression. These endpoints were assessed before and after vitamin D supplementation in BE patients with no or low grade dysplasia (LGD) for 12 weeks.

## Materials and methods

We conducted a multicenter, non-randomized interventional pilot study of vitamin D_3_ supplementation in BE patients (ClinicalTrials.gov NCT 01465113, https://clinicaltrials.gov/ct2/show/NCT01465113). The study involved 4 hospitals in the Cleveland, Ohio metropolitan area in the United States: University Hospitals Cleveland Medical Center (UHCMC), the coordinating site; Cleveland Clinic; Louis Stokes Cleveland Veterans Affairs Medical Center; and University Hospitals Ahuja Medical Center. UHCMC and Cleveland Clinic are partner institutions of the National Cancer Institute-designated Case Comprehensive Cancer Center (Case CCC). Prior to seeking Institutional Review Board (IRB) approval, the protocol underwent scientific review by the Case CCC Protocol Review and Monitoring Committee as is routinely done for cancer-related studies at our institution. This study was approved by the IRB at the coordinating center (University Hospitals Cleveland Medical Center) and participating sites (Cleveland Clinic and Louis Stokes Cleveland Veterans Affairs Medical Center).

### Study population

Potential study subjects were identified from gastroenterology clinic or from the outpatient endoscopy lab schedule. The latter were contacted prior to the date of endoscopy to ascertain interest. Patients with long-segment (≥3 cm) or short-segment (<3 cm) BE based on previous biopsies showing distal esophageal intestinal metaplasia were included. Written informed consent was obtained. Because the study was intended to focus on chemoprevention, BE patients with EAC or previous ablation therapy were excluded. BE patients with HGD treated with a shorter course of vitamin D were initially included in a separate study arm, but because of difficulty with accrual due to exclusion of prior ablation, those results are not included. Other exclusion criteria included: pregnancy, age <18 years, Child’s B cirrhosis, chronic kidney disease (creatinine ≥3.0 mg/dL), history of allergic reaction to study drugs, hypercalcemia, and inability to provide informed consent. Patients unable to abstain from NSAIDs/aspirin for the duration of the study were initially excluded; this restriction was later removed to enhance accrual. Subjects unable to abstain from NSAIDs/aspirin were instructed to stay on a stable dose from 1 week prior to the initial endoscopy until the second endoscopy. Patients who had taken >2000 IU/day of vitamin D supplementation for ≥4 weeks were excluded. Information regarding current use of calcium and vitamin D supplements was obtained. Subjects were provided gift cards at study completion as recompense for the time entailed in participation.

### Intervention

**Fig 1:**
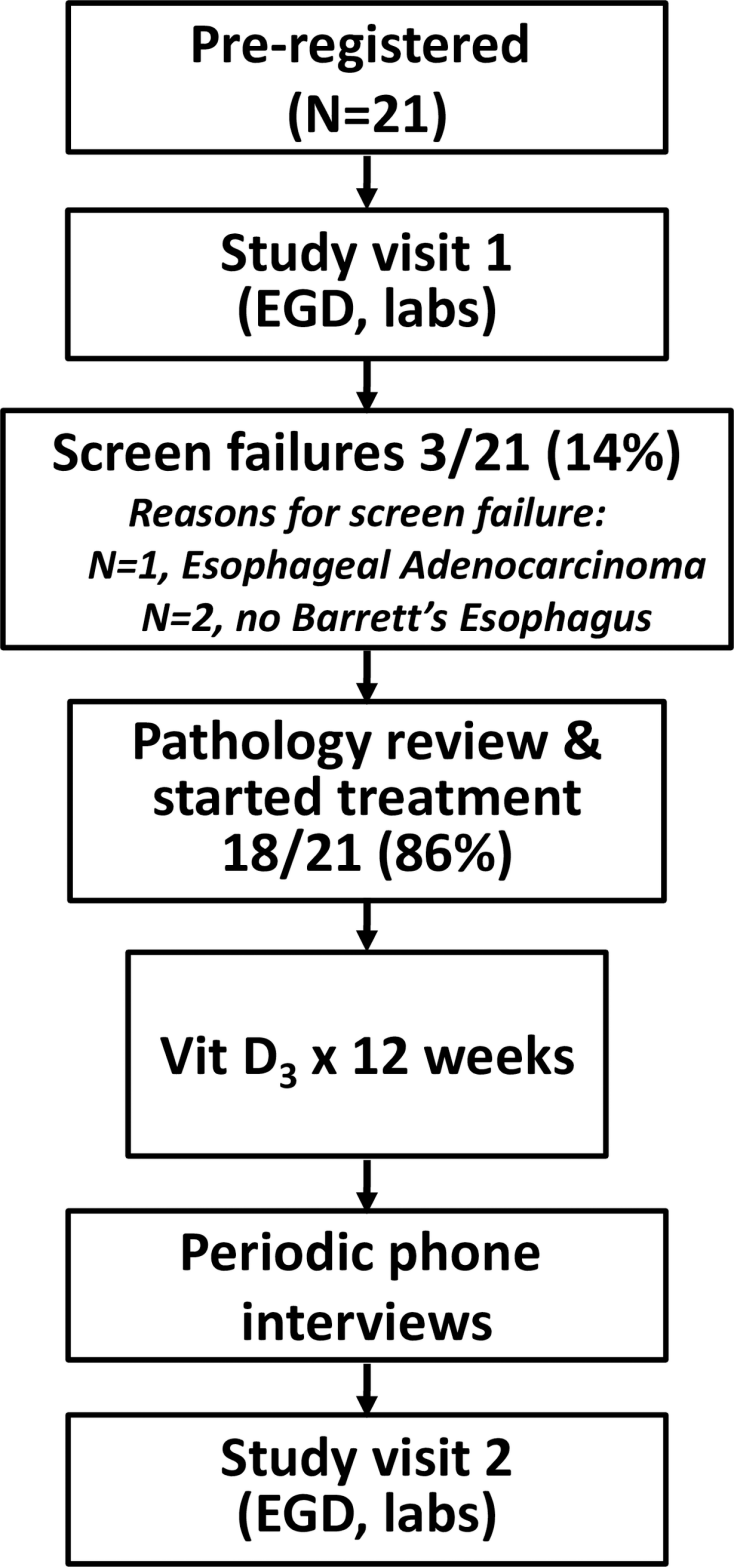
Participant flow diagram. EGD, esophagogastroduodenoscopy. Vit D_3_, vitamin D_3_.

A diagram of the flow of subjects through the trial is displayed in Fig 1. Following pre-registration (signing the informed consent form), participation began with a 28-day run-in period with a PPI (omeprazole 20 mg orally daily, or equivalent) to minimize esophagitis, which can cause histologic changes that can be mistaken for dysplasia. The run-in period was shortened in patients already taking a daily PPI. Following the run-in period, subjects underwent a baseline esophagogastroduodenoscopy (EGD) for clinical assessment of BE. During the EGD, the endoscopic appearance of salmon-colored mucosa and linear length of hiatal hernia were noted. Four quadrant surveillance biopsies were obtained in the salmon-colored segment and submitted to pathology. In addition to biopsies obtained for BE surveillance, esophageal research biopsies were obtained from salmon-colored mucosa and normal squamous mucosa ≥2 cm proximal to the squamocolumnar junction. Blood was obtained for determination of fasting serum 25-hydroxy (25-OH) vitamin D. Height and weight were noted. Waist and hip circumference were measured through a standardized protocol as previously described.[29]

Based on pathology review of the biopsies obtained for clinical care, subjects with no dysplasia or LGD were treated with vitamin D_3_ 50,000 IU orally weekly for 12 weeks. This intervention was based on dosing commonly used for vitamin D deficiency and provides a total 600,000 IU dose, which appears to be the minimal dose that most effectively achieves a 25-OH vitamin D level ≥30 ng/mL.[30] Weekly dosing was chosen over daily dosing for convenience; a prior study found that post-supplementation 25-OH vitamin D levels did not differ significantly among subjects receiving daily, weekly, or monthly regimens providing the same total dose.[31] Following supplementation with Vitamin D_3_, subjects completed a second study visit including repeat blood sampling for fasting serum 25-OH vitamin D and a repeat EGD during which research biopsies were obtained. Study drug compliance was assessed using pill counts and medication diaries. In addition, telephone interviews were conducted 2, 6, and 10 weeks after the baseline study endoscopy to assess compliance and monitor for adverse events.

### Sample processing and analysis

#### Tissue samples

Up to 6 biopsies from salmon-colored mucosa and up to 6 biopsies from normal esophageal squamous mucosa were obtained for research purposes at each endoscopy. Research mucosal biopsies were immediately snap frozen in cryogenic vials on dry ice or liquid nitrogen in the endoscopy lab during the EGD and stored at -80°C until ready for processing. Tissue samples were fixed in formalin, embedded in paraffin, sectioned, and stained for hematoxylin and eosin (H & E) in the Case CCC Tissue Procurement and Histology Core Facility. Immunohistochemical staining for 15-PGDH in intestinal metaplasia samples was also performed in the Case CCC Tissue Procurement and Histology Core Facility as detailed in Supplementary Methods. Slides were reviewed for histology and immunohistochemical staining for 15-PGDH by an expert gastrointestinal pathologist (J.E.W.) who was blinded to vitamin D treatment status (pre- versus post-supplementation). Immunohistochemical staining for 15-PGDH was graded as weak/negative, moderate, or high.

#### RNA isolation

RNA was isolated from frozen BE and normal squamous mucosal specimens in 3 batches using the mirVana miRNA Isolation Kit (Life Technologies, Carlsbad, CA) with modifications as detailed in Supplementary Methods. RNA concentration was determined using a NanoDrop-1000 Spectrophotometer (NanoDrop, Wilmington, DE).

#### Gene expression microarrays

Microarray expression analysis was carried out in the Case CCC Gene Expression and Genotyping Facility (GEGF). During sample preparation and processing, GEGF staff were blinded to vitamin D treatment status (pre- versus post-supplementation). Sample preparation is further detailed in Supplementary Methods. The Affymetrix GeneChip Human Gene 1.0 ST Array contains 32,321 probesets covering an estimated 28,869 transcripts;[32] the Affymetrix platform uses multiple probes (average is 26) to quantitate each individual transcript. The probeset for 15-PGDH has 28 unique probes arrayed along the length of the transcript. Processed arrays were scanned in the Affymetrix GeneChip Scanner 3000 7G with Autoloader for image acquisition. Because acquisition of normal esophageal samples was added to the protocol after study activation, there were 70 microarray samples (36 intestinal metaplasia samples and 34 normal squamous samples).

A quality control check was performed through Affymetrix’s Expression Console, where it was determined that 67 of 70 samples passed standard acceptable limits and were held within acceptable boundaries according to manufacturer default standards. Three samples displayed a Positive versus Negative Area Under the Curve (AUC) >2 Standard Deviations below the average and were eliminated along with their matched pairs. The remaining 64 samples, consisting of 34 intestinal metaplasia samples and 30 normal squamous samples, displayed an AUC between 0.65 and 0.85 and were retained in the data set for further analysis. Robust Multi-Chip Analysis (RMA) was used to normalize all expression values. Affymetrix probe set IDs were mapped to gene names using the HuGene-ST 1.0 NetAffx annotation file provided on the Affymetrix website.

#### Serum

Serum was extracted and stored at -80°C until analysis. Serum was analyzed for 25-OH vitamin D levels in the UHCMC Core Laboratory using a chemiluminescence immunoassay (LIAISON assay, DiaSorin, Saluggia, Italy). The inter assay coefficient of variation for the 25-OH vitamin D assay was 11.2% at a concentration of 14.5 ng/mL and 10% at a concentration of 48.2 ng/mL.

#### Drug source, storage and dispensing

Vitamin D_3_ 50,000 IU capsules were obtained from ProHealth, Inc. (Carpinteria, CA). The Investigational Drug Pharmacy at each institution stored and dispensed study drug. Study drug was provided to the subject in person or mailed to the subject.

### Statistical considerations and analysis

The initial primary objective was to detect a ≥50% increase in 15-PGDH mRNA levels with vitamin D supplementation. At the time the study was designed, data regarding 15-PGDH levels by real time RT-PCR were available in normal colon tissue but not BE, although expression of 15-PGDH in BE by IHC appeared comparable to colonic expression. Based on previous data regarding mean colonic 15-PGDH levels from 91 subjects, we estimated that 11 subjects would be needed to achieve 80% power, assuming a 20% dropout rate, a significance level of 0.05, and standard deviation of differences of 32 based on a paired t-test.

We performed simple descriptive statistics on baseline characteristics to define the study population. Categorical variables were assessed using chi-square tests or Fisher’s exact tests. For these tests, a p-value <0.05 was considered significant.

Paired t-tests were used to compare gene expression levels by pair and serum 25-OH vitamin D levels before and after vitamin D supplementation. We looked for significant changes in gene expression after vitamin D supplementation within 2 sets of paired samples: intestinal metaplasia and normal squamous esophagus samples. For evaluation of gene expression, a paired t-test raw p-value <0.001 (to account for multiple testing) was considered significant. Probes with raw p-values of <0.001 from paired t-tests were evaluated in a pathway analysis using QIAGEN’s Ingenuity^®^ Pathway Analysis (IPA^®^, QIAGEN Redwood City, www.qiagen.com/ingenuity) to identify associated functions, metabolic pathways, and signaling pathways.

Statistical analysis was conducted using R version 3.1.1 (R Foundation for Statistical Computing, Vienna, Austria) and SAS software version 9.4 (Cary, NC).

## Results

Twenty-one patients were enrolled among all sites between June 2010 and September 2013, including 3 screen failures who did not receive vitamin D supplementation (Fig 1) and 18 subjects (88%) with no dysplasia (n = 13) or LGD (n = 5) who completed the study. Baseline characteristics of subjects completing the study are presented in Table 1. Consistent with a typical BE population, study patients were older with a mean age of 64.0 years and predominantly male (78%, n = 14). 15 patients had long-segment BE, and 3 had short-segment.

**Table 1:** Baseline characteristics of participants completing the study.

	BE with no dysplasia or LGD (n = 18)
Mean age in years (SD)	64 (9.7)
Male gender, n (%)	14 (78)
Long-segment BE, n (%)	15 (83)
Mean BMI in kg/m^2^ (range)	28.9 (21.9–35.9)
Median waist-hip ratio*	0.98

BE, Barrett’s Esophagus.

LGD, Low Grade Dysplasia.

SD, Standard Deviation.

BMI, Body Mass Index.

*Among 14 subjects with hip and waist circumference measurements.

Among 18 subjects completing the study, 10 were not taking vitamin D prior to study enroll2ment, and 8 were on a multivitamin and/or a low dose vitamin D supplement. Four of 8 subjects (50%) who were taking a NSAID/aspirin prior to enrollment remained on the medication during the study. The remaining 10 subjects did not take NSAIDs/aspirin before enrollment or during study participation. Out of 18 subjects completing the study, 17 (94%) were on at least daily PPI prior to study enrollment and had a shortened run-in period; 1 subject was not on PPI prior to study enrollment and was provided omeprazole during the study.

### Adherence, adverse events, and protocol deviations

Vitamin D supplementation was generally well tolerated, with all patients receiving vitamin D supplementation completing the study protocol. Among 18 subjects, 1 returned 1 dose of 50,000 IU of vitamin D_3_ at the follow-up visit; this subject’s data were included in the analysis. One subject reported mild nausea 24–48 hours after taking the vitamin D supplement during the first 4 weeks of vitamin D supplementation; this resolved without intervention, and the subject remained in the study. Another subject reported flatulence related to omeprazole. One subject rescheduled the follow-up EGD and was treated with an additional 2 doses of vitamin D supplementation.

Three non-study-related serious adverse events occurred. 1) One subject was noted to have increased secretions from the endotracheal tube while undergoing baseline EGD under general anesthesia, and was briefly hospitalized for pneumonia after the procedure. 2) Another subject was hospitalized for atrial fibrillation with rapid ventricular response after signing the informed consent form, resulting in a delay in undergoing the baseline EGD. The subject subsequently completed the study protocol without further incident. 3) One subject had a dental abscess and root canal during the treatment phase of the study.

### Effect of vitamin D supplementation on vitamin D status

**Fig 2:**
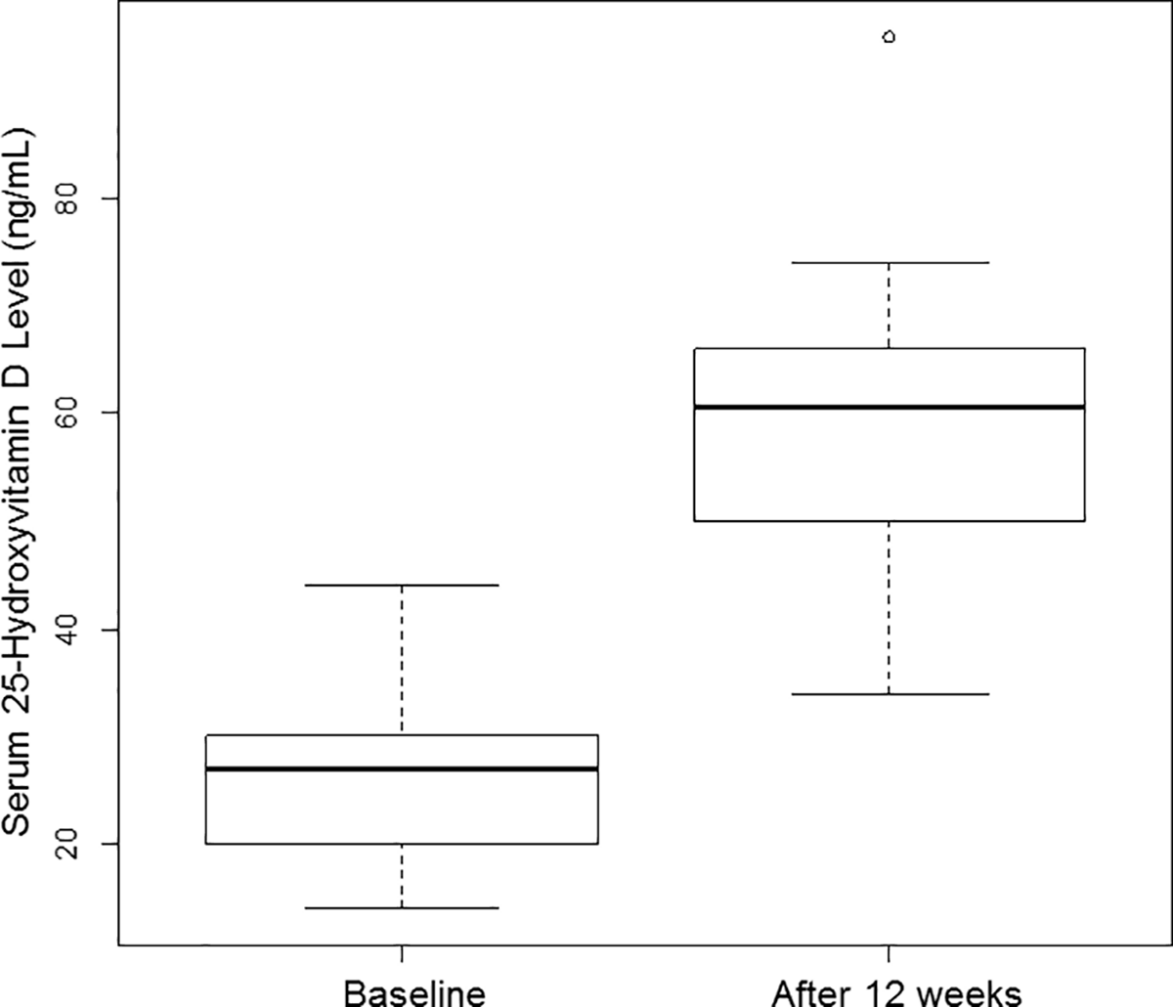
Vitamin D status at baseline and after supplementation.

Baseline serum 25-OH vitamin D levels ranged from 14.1 ng/mL (deficient) to 44.0 ng/mL (sufficient) with a median level of 27.1 ng/mL (insufficient; normal ≥30 ng/mL and insufficient 21–29 ng/mL[33]). Vitamin D status at baseline and after supplementation with vitamin D is shown (Fig 2). The median increase in serum 25-OH vitamin D level was 31.5 ng/mL which was statistically significant (p<0.0001 for paired t-test). The median 25-OH vitamin D level after supplementation was 60.7 ng/mL (range 34.0–94.9 ng/mL).

### Effect of vitamin D supplementation on gene expression, histology, and 15-PGDH immunohistochemistry

**Fig 3:**
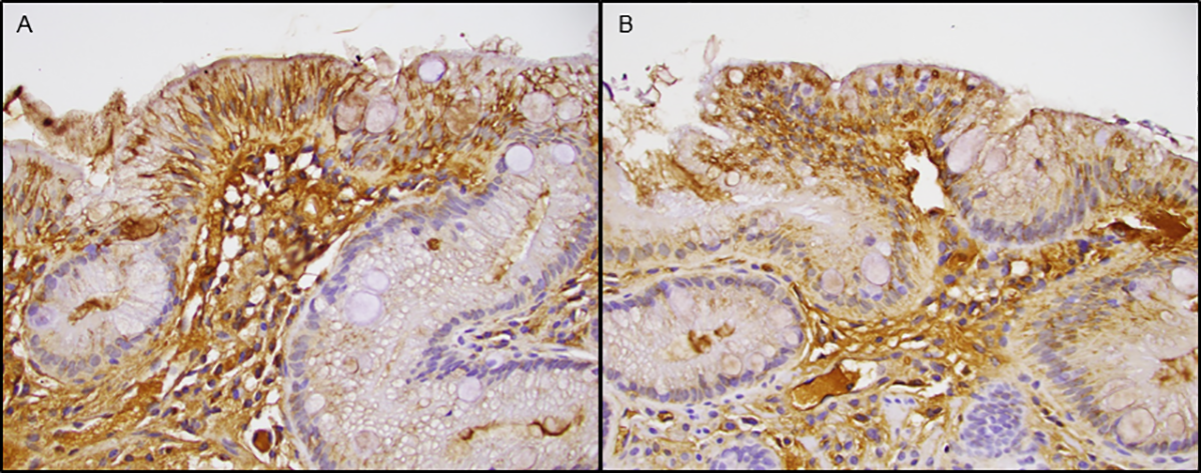
Immunohistochemistry of Barrett’s esophagus for 15-PGDH before and after Vitamin D supplementation. (A) 15-PGDH immunostaining prior to vitamin D supplementation. (B) 15-PGDH immunostaining after vitamin D supplementation within the same individual. Note no difference in staining between these two samples.

Changes in gene expression were evaluated after vitamin D supplementation within samples from BE mucosa and within normal squamous samples. After review of quality control parameters, microarray data from 17 paired BE samples and 15 paired normal squamous samples were evaluated. 17 subjects before and after vitamin D supplementation were evaluable for the primary endpoint. Based on gene expression microarrays, no significant change in expression in 15-PGDH (the primary endpoint) was observed within intestinal metaplasia or normal squamous samples. Only 1 gene within the intestinal metaplasia samples and 9 genes within the normal squamous samples were associated with a paired t-test raw p-value <0.001. However, examination of patient-level changes in expression for these genes revealed that none were associated with a unidirectional change in expression; i.e., expression decreased in some subjects but increased in others. Moreover, the associated changes were <1.5-fold, which is lower than microarray technology has the ability to reliably capture. Of note, these genes are not generally known to directly interact with vitamin D receptor. The 9 genes in normal squamous samples with a paired t-test raw p-value <0.001 were further investigated in a pathway analysis, but no association with any particular pathway or cellular process was identified. No consistent beneficial or detrimental effect of vitamin D supplementation on histology was observed. Among 7 paired intestinal metaplasia samples in which 15-PGDH staining by immunohistochemistry was assessed, 5 had no change in 15-PGDH expression with vitamin D supplementation. Representative images displaying immunohistochemical staining for 15-PGDH before and after vitamin D supplementation within an individual are shown (Fig 3).

## Discussion

This pilot study assessed the effects of vitamin D supplementation on 15-PGDH expression, vitamin D status, and global gene expression in BE. Despite significant changes in serum 25-OH vitamin D levels in patients supplemented with 12 weeks of vitamin D_3_, we demonstrated no significant change in gene expression (including 15-PGDH) with vitamin D supplementation. Examination of a few genes that modestly changed with vitamin D supplementation within normal squamous samples did not reveal modulation of common pathways or cellular processes. Evaluation of gene expression in BE mucosa compared to normal squamous samples did demonstrate changes in expression of genes previously reported to be differentially expressed in other articles focused on this topic,[34–36] supporting our sampling methodology. Our results suggest that BE does not respond to treatment with vitamin D supplementation, at least at doses typically used for vitamin D deficiency.

The negative results of this study are somewhat surprising given translational and clinical data that support some rationale for treating BE with vitamin D. 15-PGDH is downregulated in BE with HGD compared with non-dysplastic BE,[22] and vitamin D has been shown to induce 15-PGDH in other cancers.[23, 24] Vitamin D receptor is expressed in normal esophageal mucosa, BE, EAC, and EAC cell lines.[34, 37–39] VDR expression is upregulated in BE compared with normal esophageal mucosa,[34, 38] suggesting that BE may be more sensitive than normal esophageal mucosa to the effects of calcitriol, the active form of vitamin D. Recent data also suggest that genetic variations in VDR are linked to reduced EAC risk.[40] Clinically, low vitamin D levels have been associated with insulin resistance and metabolic syndrome,[41] both of which are common in BE patients.[29, 42] Moreover, vitamin D deficiency is associated with an increased risk, as well as worse outcomes, in other cancers.[43–46] However, it is unknown whether vitamin D status affects development of Barrett’s esophagus.

In the current study, the mean 25-OH vitamin D level prior to supplementation was 27 ng/mL, consistent with vitamin D insufficiency. These results are similar to recent findings by Thota et al. reporting a mean 25-OH vitamin D level of 72 nmol/L (29 ng/mL) in a registry of 429 BE patients.[47] In that study, there was no association between vitamin D status and incidence or prevalence of HGD/EAC. An older prospective study demonstrated an association between low vitamin D levels and increased risk for esophageal cancer, not further delineated by histologic subtype.[28] Although a more recent case-control study showed no association between circulating 25-OH vitamin D levels and any upper gastrointestinal tract cancer, EAC comprised <10% of cases in the study.[48] A case-control study from Northern Ireland found an inverse relationship between dietary vitamin D intake and EAC risk, but did not adjust for vitamin D supplementation and did not include serum 25-OH vitamin D levels.[49] A recently published systematic review and meta-analysis did not find a consistent association between vitamin D exposure and esophageal neoplasia.[50]

We chose cholecalciferol (vitamin D_3_) as the form of vitamin D used in this study due to its potency compared with ergocalciferol (vitamin D_2_).[51] Cholecalciferol must be converted through hydroxylation to the active form of vitamin D, 1,25 dihydroxyvitamin D (1,25(OH)_2_ vitamin D), also known as calcitriol. Calcitriol directly or indirectly controls over 200 genes that affect proliferation, apoptosis, and differentiation.[33] The last hydroxylation step is catalyzed by CYP27B1, a cytochrome P450 enzyme with 1 α-hydroxylase activity predominantly expressed in the renal tubules. Extrarenal expression of CYP27B1, which in the gastrointestinal tract has been reported in the colon and stomach,[52, 53] supports the notion that regulation of local concentrations of calcitriol may affect key cellular functions including growth and differentiation in a tissue-specific manner. To our knowledge, CYP27B1 expression in the esophagus has not previously been reported, and one potential explanation for the lack of Vitamin D effect in BE may be that CYP27B1 is not expressed in the esophagus.

Our results may have been biased towards the null as a result of the use of vitamin D supplements by some study subjects prior to enrollment. The use of multivitamins, which contain a low dose (400 IU) of vitamin D, likely had a negligible impact on serum 25-OH vitamin D levels based on a previous study.[54] Vitamin D supplements are commonly used. Indeed, the ongoing VITAL study (Vitamin D and Omega-3 Trial, clinicaltrials.gov NCT01169259), a large trial assessing the impact of 2,000 IU of daily vitamin D_3_ or omega-3 fatty acid supplementation on cancer, heart disease, and stroke, allows intake of up to 800 IU daily of non-study vitamin D supplements.[55] However, it is possible that subjects could have received treatment for vitamin D deficiency at some point prior to enrollment. In the current study, the upper range of baseline serum 25-OH vitamin D levels was 44.0 ng/mL. Moreover, to improve adherence, the duration of supplementation was 12 weeks based on dosing commonly used for vitamin D deficiency as well as previous BE chemoprevention trials using aspirin and metformin.[15, 56].

Chemoprevention is a potentially advantageous strategy that warrants investigation because BE is the only known precursor to EAC, which has risen dramatically in incidence and carries a poor prognosis. The importance of EAC prevention is reflected in gastrointestinal society guidelines which recommend ablation over surveillance for BE with HGD.[57, 58] Despite their effectiveness, ablative modalities require multiple treatments (and hence repeat endoscopies) and are not 100% effective.[7] Although vitamin D supplementation in comparison is well-tolerated and economical, the study’s results unfortunately do not support a role for vitamin D supplementation in EAC chemoprevention. A larger sample size may have been needed to see an effect; the sample size calculation was based on colonic 15-PGDH mRNA levels, the best data available at the time the study was designed. Alternatively, it is possible that higher post-supplementation serum 25-OH vitamin D levels would have been needed to see an effect at the tissue level.

Although this pilot study did not find significant changes in gene expression with vitamin D supplementation for BE, the results add to existing chemoprevention studies in this field.[15, 16, 56] To our knowledge, no prior studies have evaluated the *in vivo* effects of vitamin D supplementation on BE. Study participants were recruited from multiple centers. The study was conducted in northeast Ohio, an area in the United States with higher risk for vitamin D deficiency due to its northern climate and latitude. The study was limited by its non-randomized, non placebo-controlled design. However, the endpoints were objective measures, and the paired design removed bias due to inter-subject variability. Recruitment was challenging due to the requirement for subjects to undergo a second EGD for research purposes; the initial exclusion of patients unable to abstain from NSAIDs during the study was lifted to improve accrual, but NSAID use could have impacted the results. Although our results may not necessarily be generalizable due to the limited sample size, the baseline characteristics of our participants (male predominance, older age, and white race) reflect the typical BE population.

## Conclusions

In conclusion, supplementation with vitamin D_3_ 50,000 IU weekly in conjunction with daily PPI for up to 12 weeks was well tolerated in BE patients with or without dysplasia. However, this regimen did not lead to improvements in gene expression. Despite a potential association between vitamin D deficiency and esophageal cancer risk, the results of this study do not support the use of a 12-week course of vitamin D supplementation for EAC chemoprevention. Nonetheless, given the dismal outcomes associated with EAC, additional chemoprevention studies are warranted. Future studies could also evaluate the impact of vitamin D status on Barrett’s esophagus risk or assess the effect of changes in 15-PGDH expression on esophageal adenocarcinoma risk within individuals over time.

## Supporting information

S1 File Supplementary methods. (DOC)

S2 File TREND checklist. (PDF)

S3 File Protocol. (DOC)
