# Supplementary material for: A nonrandomized trial of vitamin D supplementation for Barrett’s esophagus
Source: PLoS One. 2017 Sep 18;12(9):e0184928. doi: 10.1371/journal.pone.0184928 (PMC5602627; doi:10.1371/journal.pone.0184928)
Supplement: S1 File — (DOC) [file pone.0184928.s001.doc]

Supplementary Methods

RNA isolation

RNA isolation using the mirVana miRNA Isolation Kit (Life Technologies, Carlsbad, CA) was performed with the following modifications. Frozen specimens were placed in 2 ml-capacity round-bottom microfuge tubes along with 1 ml of mirVana lysis buffer. A single large steel ball was placed inside each tube. Tubes were loaded onto the TissueLyser II (Qiagen, Valencia, CA) and the tissues were disrupted by subjecting the tubes to 2 cycles of 2 minutes at 180 cycles/minute of high speed shaking.  Significant foaming occurred. Tubes were set on ice for 30 minutes to allow the foam to abate. The lysate was then removed by pipet to a fresh microfuge tube and further processed according to the mirVana manufacturer’s instructions. Each batch included pre- and post-vitamin D samples from a given patient. RNA was eluted from the spin columns using 30 μl of RNase free water.

Immunohistochemistry

Immunohistochemical analysis for 15-PGDH was performed in the Case CCC Tissue Procurement and Histology Core Facility. Tissue sections were deparaffinized in xylene and rehydrated through graded ethanols to dH_2_O. Antigen retrieval was performed using a pressure cooker and Citrate retrieval buffer (pH 6.0). Endogenous peroxidase activity was quenched using 3% hydrogen peroxide. Background noise was suppressed with a casein based blocking reagent. 15-PGDH staining was performed using a mouse monoclonal antibody developed in Dr. Sanford Markowitz’s laboratory diluted at 1:800 and incubated overnight at 4⁰C. Detection of antibodies was accomplished using horseradish peroxidase-conjugated polymer technology (BioCare Medical) and visualized with 3,3’-Diaminobenzidine (DAB) chromogen. Nuclear counterstaining was done using Hematoxylin. Colon mucosa was used as a positive control for 15-PGDH.
