# Supplementary material for: A nonrandomized trial of vitamin D supplementation for Barrett’s esophagus
Source: PLoS One. 2017 Sep 18;12(9):e0184928. doi: 10.1371/journal.pone.0184928 (PMC5602627; doi:10.1371/journal.pone.0184928)
Supplement: S3 File — (DOC) [file pone.0184928.s003.doc]

Study Title: Effect of Vitamin D Supplementation on 15-Prostaglandin Dehydrogenase Expression in Barrett’s Esophagus

## Background and Rationale

Esophageal adenocarcinoma (EAC) has dramatically tripled in incidence in the United States over the last 30 years and is strongly linked to its precursor metaplastic lesion, Barrett’s esophagus (BE), gastroesophageal reflux disease (GERD), and obesity. Given the overall poor prognosis associated with esophageal cancer, with a 5-year relative survival rate of <20% in the United States, inhibiting the progression of BE to esophageal adenocarcinoma would be ideal.

Chemoprevention is an attractive strategy for prevention of esophageal adenocarcinoma. In a rat model of Barrett’s esophagus, non-steroidal anti-inflammatory drugs (NSAIDs) reduced the risk of developing EAC. NSAIDs work by inhibiting prostaglandins through suppression of the two cyclooxygenase enzymes, COX-1 and COX-2; the latter has been shown to be overexpressed in Barrett’s esophagus and esophageal adenocarcinoma. Taken together, these studies suggest a role for the cyclooxygenase-2 (COX-2) pathway in EAC pathogenesis. Although celecoxib, a COX-2 inhibitor, was not found to prevent progression of BE to EAC, further studies in this area are warranted.

15-prostaglandin dehydrogenase (15-PGDH) is a tumor suppressor involved in degradation of intracellular prostaglandin E_2_ (PGE_2_), a prostaglandin in the COX-2 pathway that promotes tumorigenesis. 15-PGDH expression is decreased in Barrett’s esophagus with dysplasia. Although 15-PGDH expression by RT-PCR has not been evaluated in Barrett’s esophagus, data from colonic mucosa suggest a close correlation between levels by IHC and RT-PCR. This new data lends itself to therapeutic targets for EAC chemoprevention. Calcitriol, the active form of vitamin D, has been shown to induce 15-PGDH in prostate cancer cells, thereby decreasing pro-inflammatory prostaglandins. It is being studied as a therapeutic agent in prostate cancer. Low vitamin D levels have been associated with an increased risk for esophageal cancer, although no distinction was made between esophageal squamous cell carcinoma and adenocarcinoma. If vitamin D can induce 15-PGDH in the esophagus, it could potentially be used as a chemopreventive agent in Barrett’s esophagus.

As an initial study to evaluate the effects of vitamin D in Barrett’s esophagus, this project aims to assess 15-PGDH levels, vitamin D status and global gene expression in Barrett’s mucosa before and after vitamin D supplementation.

Primary Objective

To determine whether vitamin D supplementation with induces 15-Prostaglandin dehydrogenase expression in Barrett’s esophagus

Secondary Objectives

To determine the effects of vitamin D supplementation on vitamin D status and global gene expression

## Patient Selection

## Inclusion Criteria

1. Short-segment or long-segment Barrett’s esophagus confirmed by prior upper endoscopy showing salmon-colored distal esophageal mucosa and biopsies revealing intestinal metaplasia with goblet cells
2. Age ≥18 years
3. Willing to donate 90 mL of blood and endoscopic mucosal biopsies for research
4. Subjects may be taking calcium supplements or have previous history of hypercalcemia.
5. Subjects may have a history of prior malignancy except for esophageal adenocarcinoma.

Exclusion Criteria

1. Pregnancy
2. Age <18 years
3. Chronic liver disease (Child’s B cirrhosis)
4. Chronic kidney disease (creatinine ≥ 3.0 mg/dL)
5. Esophageal adenocarcinoma
6. Allergy to omeprazole or vitamin D
7. Unable or unwilling to provide informed consent
8. Hypercalcemia
9. Previous ablation for BE
10. Patients on >2000 IU/day of vitamin D supplementation for ≥4 weeks

Intervention

Potential subjects for the study will be identified from patients with previously diagnosed Barrett’s esophagus and may be contacted by mailings, phone calls, or approached in clinic to ascertain interest in participation. Potential subjects will be given the opportunity to review the informed consent form. If they are not considered medically essential, NSAIDs may be held during the study due to their effects on the cyclooxygenase-2 pathway, the pathway of interest in this study. Patients on chronic NSAIDs which cannot be discontinued can be in the study but must stay on a stable dose for the duration of the study (from 1 week prior to initial endoscopy until 2^nd^ endoscopy).

Informed consent will be obtained prior to a 28-day run-in phase during which subjects are treated with a proton pump inhibitor (omeprazole 20 mg po q day or an equivalent dose of another proton pump inhibitor). This run-in phase – the interval between signing the informed consent form and the next phase of the study, an upper endoscopy – is not necessary in patients who have already been on a proton pump inhibitor for at least 28 days and can be shortened accordingly for patients recently started on a proton pump inhibitor before they signed informed consent. The purpose of the run-in phase is to minimize esophagitis, which can cause histologic changes that can be confused with dysplasia. The proton pump inhibitor will be continued for the duration of the study.

After the run-in phase, subjects will undergo an upper endoscopy for Barrett’s surveillance as part of routine clinical care. At the time of endoscopy, in addition to large cup forceps biopsies obtained as part of standard care, research biopsies will be obtained for the study for measurement of mucosal levels of 15-PGDH. Up to 6 research biopsies will be obtained from Barrett’s mucosa (2 for each cm of length of Barrett’s esophagus, up to 3 cm). Up to 6 additional research biopsies will also be obtained from normal upper tract epithelium. Subjects will have baseline fasting serum 25-OH vitamin D levels determined from blood drawn on the day of the upper endoscopy. Information will be obtained from the patient regarding current intake of vitamin D or calcium supplements. All medications (prescription and over-the-counter), vitamin and mineral supplements, and/or herbs taken by the participant will be documented on the concomitant medication CRF and will include: 1) start and stop date, dose and route of administration, and indication.

In the uncommon event that biopsies are read by pathology as “indefinite for dysplasia” or “indeterminate for dysplasia,” the subject will be treated similarly to BE subjects with low grade or no dysplasia. These patients (indefinite for dysplasia, LGD, or no dysplasia) will take vitamin D_3_ 50,000 IU once a week for 12 weeks following the upper endoscopy. Study drugs will be dispensed by the Investigational Pharmacy. Vitamin D_3_ is preferred to vitamin D_2_ (ergocalciferol) because it is more potent. Following vitamin D_3_ supplementation, all subjects will undergo a repeat upper endoscopy. At that time, additional large cup forceps biopsies will be obtained for measurement of post-treatment mucosal levels of 15-PGDH. Up to 6 research biopsies will be obtained from Barrett’s mucosa (2 for each cm of length of Barrett’s esophagus, up to 3 cm). Up to 6 additional research biopsies will also be obtained from normal upper tract epithelium. At that time, serum 25-OH vitamin D will be drawn, and compliance with vitamin D supplementation will be assessed using pill counts and a medication diary.

Treatment Delays and Dose Reductions/Modifications

The dose of vitamin D_3_ proposed for this study is available without a prescription. The doses/duration planned for the study pose minimal risk to subjects.

Toxicity monitoring
Adverse effects from vitamin D_3_ intake are exceedingly rare; evidence from clinical trials suggests that prolonged intake of up to 10,000 IU per day of vitamin D_3_ even in individuals with a high physiologic levels of vitamin D, is not associated with adverse effects. Hypercalcemia is associated with excessive doses of vitamin D (more than 50,000 IU per day). The doses and duration of vitamin D_3_ proposed for the study pose minimal risk.

Subjects will be assessed for side effects to study medications/supplements via telephone interviews conducted on Days 14, 42, and 70 of the study.

Removal from Study

Subjects may be removed from the study if they develop side effects related to study supplements that are felt to be harmful, are not following the rules of the study, or if funding for the study is stopped.

Adverse Event Reporting

Adverse events will be reported to the Designated Safety Officer, Jeffry Katz, M.D., a staff physician in the UHCMC Division of Gastroenterology. Each subject will be evaluated for any adverse events, which are defined as expected side effects of a serious nature, or unexpected side effects/events regardless of severity. All adverse events will be graded as mild, moderate, or severe. Any severe and/or unanticipated adverse event will be immediately reported to the safety officer and Cancer Center Data and Safety Toxicity Committee. All other adverse events will be reported in a timely fashion to the safety officer and Cancer Center Data and Safety Toxicity Committee, preferably within 2 weeks of the date of the event.

Data Safety Monitoring Plan

This protocol will adhere to the policies of the Cancer Center Data and Safety Monitoring Plan, version 3 guidelines in accordance with NCI regulations. The Data and Safety Toxicity Committee will review all serious adverse events and toxicity reports as well as annual reviews.

In the case of hemorrhage or perforation related to upper endoscopy (known risks associated with the procedure), the trial will be stopped pending review by the Designated Safety Officer, Jeffery Katz, M.D. and the Data and Safety Toxicity Committee. The Case Comprehensive Cancer Center Data and Safety Toxicity Committee will provide the safety monitoring for this protocol.

Measurement of Effect

The primary objective of this study is to determine whether vitamin D supplementation induces 15-PGDH expression in Barrett’s esophagus. Secondary objectives assess whether vitamin D supplementation affects global gene expression in Barrett’s esophagus. Research biopsies will be obtained at the time of the first and follow-up endoscopy. Two large cup forceps biopsies for each centimeter of Barrett’s mucosa, up to a maximum length of 3 cm, will be obtained for the study for measurement of mucosal levels of 15-PGDH. Up to 6 additional research biopsies will also be obtained from normal upper tract epithelium. Biopsies will be snap frozen in cryogenic vials using dry ice or liquid nitrogen (ideally 1 sample per vial) and delivered to Dr. Sanford Markowitz’ laboratory for processing.

mRNA will be extracted from biopsy tissue to assess 15-PGDH levels and global gene expression. Global gene expression in response to vitamin D supplementation will be assessed using gene expression arrays. Additionally, biopsies will be fixed in formalin, embedded in paraffin, section, and stained for hematoxylin and eosin for review of histology. Immunohistochemical staining for 15-PGDH will be performed in Barrett’s mucosa samples.

Thirty cc (two 10 ml red top for serum and one 10 ml purple top tube for plasma) of blood will be collected from all patients enrolled in the study at the time of initial and follow-up endoscopy by a research nurse. Fasting serum 25-OH vitamin D will be measured based on blood drawn at the time of endoscopy, before and after vitamin D supplementation. Serum 25-OH vitamin D levels will be sent to the University Hospitals Cleveland Medical Center Laboratory.

Drug Information (Dose and Mode of Administration)

Omeprazole 20 mg capsules po; cholecalciferol 50,000 IU capsules po

Sample Size Calculation

The null hypothesis is that there is no difference in esophageal mucosal 15-PGDH levels before and after vitamin D supplementation. The alternative hypothesis is that 15-PGDH levels increase by at least 50% after vitamin D supplementation.

15-PGDH levels have been assessed in Barrett’s mucosa by immunohistochemistry, but not by RT-PCR. Colonic 15-PGDH levels by RT-PCR correlate well with immunohistochemistry data. Previous work in Dr. Markowitz’ lab looked at 15-PGDH levels by RT-PCR in normal colon mucosa (unpublished data). Assuming a 50% increase in 15-PGDH levels from the mean level in normal colon of 91 subjects and a significance level of 0.05 using a paired t-test, the sample size required to achieve a power of 80% or 90% is shown in the table below. We used calculated mean difference and standard deviation of differences from Dr. Markowitz’s data in colon. We assumed that all tests are two-sided.

Our study called for 11 subjects to achieve 80% power, assuming a 20% dropout rate, a significance level of 0.05, and standard deviation of differences of 32.

Statistical analysis

We will perform simple descriptive statistics on baseline characteristics such as age, gender, and race to define the study population. Gene expression levels before and after vitamin D supplementation will be compared using paired t-tests. Categorical variables will be assessed using chi-square tests or Fisher’s exact tests.

References

1. Devesa SS, Blot WJ, Fraumeni Jr. FJ. Changing patterns in the incidence of esophageal and gastric carcinoma in the United States. Cancer. 1998;83:2049-53.

2. Lagergren J, Bergstrom R, Lindgren A, Nyren O. Symptomatic gastroesophageal reflux as a risk factor for esophageal adenocarcinoma. New Engl J Med. 1999;340(11):825-31.

3. Hampel H, Abraham NS, El-Serag HB. Meta-Analysis: Obesity and the risk for gastroesophageal reflux disease and its complications. Ann Intern Med. 2005;143:199-211.

4. Buttar NS, Wang KK, Leontovich O, Westcott JY, Pacifico RJ, Anderson MA, et al. Chemoprevention of esophageal adenocarcinoma by COX-2 inhibitors in an animal model of Barrett's esophagus. Gastroenterology. 2002;122(4):1101-12.

5. Wilson K, Fu S, Ramanujam K, Meltzer S. Increased expression of inducible nitric oxide synthase and cyclooxygenase-2 in Barrett's Esophagus and associated adenocarcinomas. Cancer Res. 1998;58:2929-34.

6. Heath E, Canto M, Piantadosi S, Montgomery E, Weinstein W, Herman J, et al. Secondary chemoprevention of Barrett’s esophagus with celecoxib: Results of a randomized trial. J Natl Cancer Inst. 2007;99:545-57.

7. Sabo E, Meitner PA, Tavares R, Corless CL, Lauwers GY, Moss SF, et al. Expression Analysis of Barrett's Esophagus–Associated High-Grade Dysplasia in Laser Capture Microdissected Archival Tissue. Clin Cancer Res. 2008;14(20):6440-8.

8. Moreno J, Krishnan A, Swami S, Nonn L, Peehl D, Feldman D. Regulation of prostaglandin metabolism by calcitriol attenuates growth stimulation in prostate cancer cells. Cancer Res. 2005;65:7917-25.

9. Giovannucci E, Liu Y, Rimm EB, Hollis BW, Fuchs CS, Stampfer MJ, et al. Prospective study of predictors of Vitamin D status and cancer incidence and mortality in men. J Natl Cancer Inst. 2006;98:451-9.

10. Houghton LA, Vieth R. The case against ergocalciferol (vitamin D2) as a vitamin supplement. Am J Clin Nutr. 2006;84(4):694-7.

11. Vieth R. Vitamin D and cancer mini-symposium: The risk of additional vitamin D. Ann Epidemiol. 2009;19:441-5.

12. Holick MF. Vitamin D Deficiency. New Engl J Med. 2007;357(3):266-81.
